# Target-specific therapeutic assessment of repurposed drug candidates for oral lichen planus: a network pharmacology-molecular dynamics simulation guided investigation

**DOI:** 10.1038/s41405-025-00384-y

**Published:** 2025-12-19

**Authors:** Alaka Sahoo, Shasank Sekhar Swain, Satya Ranjan Singh, Atala Bihari Jena, Sudhir Kumar Paidesetty, Asim K. Duttaroy, Maitreyee Panda

**Affiliations:** 1 Department of Skin & VD, Institute of Medical Sciences and SUM Hospital, Siksha ‘O’ Anusandhan Deemed to be University, Bhubaneswar, Odisha India; 2 BRIC-Institute of Life Sciences Bioincubator, Bhubaneswar, Odisha India; 3 Department of Bioinformatics, Pondicherry University, Puducherry, India; 4 National Centre for Cell Science, Savitribai Phule Pune University Campus Ganeshkhind, Pune, India; 5 Department of Pharmaceutical Chemistry, School of Pharmaceutical Sciences, Siksha ‘O’ Anusandhan Deemed to be University, Bhubaneswar, Odisha India; 6 Department of Nutrition, Institute of Medical Sciences, Faculty of Medicine, University of Oslo, Oslo, Norway

**Keywords:** Diseases, Oral diseases

## Abstract

**Background:**

Oral lichen planus (OLP) is a chronic mucocutaneous autoimmune skin disease without a proper pathophysiology and approved therapy. As a result, several repurposed drugs have been used in clinical practice, and it remains unclear which one holds greater potential.

**Aim:**

The present study employs a network pharmacology to explore the disease biology and further investigate the target-specific binding efficacy of repurposed drugs.

**Materials and methods:**

Twenty-eight repurposed drug’s (**D1**-**D28**) efficacies against twelve targets were investigated using PyRx 0.8-AutoDock 4.2 software. Further, drug stability and reactivity were studied using molecular dynamics (MD) simulation at 200 ns, Gibbs free energy, frontier molecular orbital theory, and structural activity relationship.

**Results:**

The above computational investigation suggested betamethasone (**D2**/BETA) and triamcinolone acetonide (**D28**/TACA) are two potential drugs, predominantly demonstrating higher binding efficacy against the glucocorticoid receptor (GR). Further, MD simulation, free-energy calculation revealed that **D28/**TACA was comparatively more stable than **D2**/BETA.

**Conclusion:**

The network pharmacology explored possible drug targets for drug discovery and showed that **D28/**TACA is a more effective treatment option among repurposed drugs.

## Introduction

Oral lichen planus (OLP) is a chronic, immune-mediated inflammatory disease with a global prevalence of 0.5–2% among adults [1–4]. It is eight times more common than cutaneous lichen planus and may precede or occur alongside skin lesions [5, 6]. OLP typically affects the oral cavity, particularly the bilateral buccal mucosa, tongue, and gingiva. OLP comes in different forms, but the reticular variant is the most common in clinical practice [5, 6]. It is marked by white, lace-like sores on the mucous membranes, which usually do not cause any symptoms but can sometimes cause mild burning sensations without altering dietary habits [5–9]. However, the erosive and ulcerative variants, often affecting the tongue, are notably painful. Desquamative gingivitis occurs as an isolated manifestation in about 8% of cases [10, 11]. Despite its recognition as an autoimmune disorder, the exact etiology of OLP remains moderately understood [6, 12, 13]. The evidence points to a complex interplay of immunological and environmental factors in its pathogenesis, with ongoing research aimed at uncovering these mechanisms [14–17].

Currently, various treatment modalities and drug formulations are used for managing symptomatic OLP as per the conditions (mild, moderate, and severe) [18–20]. From therapeutic perspective, topical corticosteroids commonly used for mild cases include clobetasol propionate (CP), fluocinonide (FLOC), fluticasone (FP), triamcinolone acetate (TACA), betamethasone (BETA), and fluocinolone acetonide (FCLA) [18–20]. Topical cyclosporine or tacrolimus (CyA/TACR), in conjunction with potent corticosteroids like CP, FP, typically treat moderate cases [18–20]. Some medicines that can help with severe OLP are CyA, acitretin, azathioprine, dapsone (DDS), cyclophosphamide, hydroxychloroquine (HCQ), and thalidomide (THA) [9, 11, 21–23]. For refractory cases, biologics (adalimumab, alefacept, etanercept, guselkumab, rituximab, secukinumab, tildrakizumab, and ustekinumab) have been used to control OLP by regulating the expression of cluster of differentiation 2 (CD2), tumor necrosis factor-alpha (TNF-α), interleukin-2 (IL2), interleukin-17 (IL17), interleukin-12/23 (IL12/23), etc [11, 24, 25].

In routine clinical practice, physicians commonly use topical triamcinolone (TAC) and intralesional TAC for OLP, and for non-responders or extensive cases, oral mini-pulse therapy with BETA (twice a week) is employed to improve dietary habits [26]. However, long-term steroid use carries potential adverse effects [13, 20, 27]. Therefore, alternative physicians recommended several immunomodulatory and non-steroidal anti-inflammatory drugs like methotrexate (MTX), apremilast (APR), azathioprine, and dapsone (DDS) [3, 21, 28, 29]. Recent research suggests that putting pimecrolimus, thalidomide (THA), tacrolimus (TACR), and amlexanox (AMX) on the skin is effective as a new way to treat OLP [11, 24, 25, 29]. The limited treatment options, lack of Food and Drug Administration (FDA)-approved drugs, and insufficient understanding of the precise mechanisms make it difficult to manage OLP [9, 22, 23, 27, 30]. Dermatologists also face the challenge of selecting the most appropriate therapy for each specific case [31]. Therefore, the management of OLP urgently requires suitable therapeutic candidates as well as identification of the most potent targets to explore newer therapeutics.

This study initially explores the most possible biological targets associated with OLP through systems biology and network pharmacology approaches to be used as targets in therapeutic assessment. Using the computer-aided drug design (CADD) platform, we evaluate the target-specific therapeutic efficacy of both repurposed steroidal and non-steroidal candidates (n = 28). Briefly, we evaluate the target-specific binding efficiency and conformational stability of these drugs against 12 targets through molecular docking and molecular dynamic (MD) simulation. In addition, frontier molecular orbital (FMO) energy, drug-likeness (DL) profiles, and structure-activity relationships (SAR) of a selective steroid class of drugs are used to select the promising drug candidates for OLP.

## Material and methods

### Network pharmacology to explore disease networks and putative targets of OLP

Systems biology and network pharmacology were used to explore the disease-target network of OLP by integrating data from multiple curated datasets. For our study, DisGeNET (https://www.disgenet.com/), GeneCards (https://www.genecards.org/), and Open Targets (https://www.opentargets.org/) data sets are used to explore relevant molecular targets associated with OLP pathogenesis [32, 33]. We employed a systematic network pharmacology approach, which involved analyzing protein-protein interaction (PPI) or functional protein-associated network using STRING 12.0, bioactive target networking using Cytoscape 3.1, and conducting gene enrichment analyses (GO enrichment and the KEGG pathway) using the ShinyGO 0.80 tool [34, 35]. Based on the above investigation, six relevant genes, interleukin IL-alpha (IL1α), IL2, IL4, IL6, IL10, and IL17α, were selected based on the hub ranking degree and fold enrichment value. In addition, another six targets, tumor necrosis factor alpha 1 (TNFα1), interferon gamma (IFNγ3), nuclear factor kappa B (NF_K_B), glucocorticoid receptor (GR), heat shock protein 70 (HSP70), and toll-like receptor 7 (TLR7), were selected based on recent clinical reports towards exploring the efficacy and structure insights in a reliable and unbiased approach [36–41].

### Drug and target structure retrieval and preparation

Based on extensive clinical reports, the chemical structure of twenty-eight existing drugs (oral, injectable, topical, and other administered routes) was selected [42–45]; Table S1]. Accordingly, the three-dimensional (3D) chemical structure of the individual drug that served as the ligand structure was retrieved from the PubChem database (https://pubchem.ncbi.nlm.nih.gov/) (see Table 1 and Table S1). Further, each ligand structure was optimized using the Merck molecular force field 94 (MMFF94) before the docking study. The X-ray crystallographic 3D protein structure of twelve targets (six from network pharmacology and six from recent clinical reports) was retrieved from the protein data bank (https://www.rcsb.org/). The PDB IDs of retrieved structures are IL1α (PDB ID: 5UC6), IL2 (PDB ID: 1M49), IL4 (PDB ID: 1HIK), IL6 (PDB ID: 4O9H), IL10 (PDB ID: 1LQS), IL17α (PDB ID: 8CDG), TNFα1 (PDB ID: 1TNF), IFNγ3 (PDB ID: 1QWT), NF_K_B (PDB ID: 1SVC), HSP70 (PDB ID: 6FDT), GR (PDB ID: 4P6W), and TLR7 (PDB ID: 5GMG), respectively. Before the docking study, we remodeled the protein structure to assemble splitting amine residues, removed attached ligands, chains, and heteroatoms, and made individual grid box settings (see Table S2**)** to achieve reliable docking scores [46, 47].

**Table 1:** Molecular docking study of selected drugs (**D1-D28**) against twelve putative target enzymes associated with OLP development and progress.

Sl. No.	Based on network pharmacology						Based on recent clinical reports						Avg. score	DL score
	IL1α	IL2	IL4	IL6	IL10	IL17α	TNFα1	IFNγ3	NF_K_B	HSP70	GR	TLR7		
**D1**	−5.6	−5.5	−6.5	−6.2	−6.1	−5.5	−8.1	−5.7	−5.5	−5.3	−7.3	−8.4	−6.3	−0.57
**D2**	−7.7	−9.9	−6.4	−7.9	−6.8	−8.0	−9.9	−7.4	−7.7	−6.9	−11.4	−8.4	−8.2	1.02
**D3**	−5.9	−5.9	−6.3	−6.1	−6.2	−6.8	−7.4	−7.2	−5.6	−6.0	−8.8	−7.5	−6.6	−0.81
**D4**	−5.3	−5.2	−5.4	−5.6	−5.9	−5.2	−6.9‘	−6.3	−5.2	−5.1	−6.8	−6.1	−5.8	1.97
**D5**	−6.5	−6.4	−6.9	−6.5	−6.7	−6.6	−9.2	−6.9	−6.1	−5.6	−6.8	−8.3	−6.9	1.07
**D6**	−5.9	−5.7	−5.3	−5.9	−5.9	−7.5	−6.7	−6.4	−5.8	−5.5	−7.0	−7.3	−6.2	−0.86
**D7**	−5.8	−5.7	−5.8	−5.9	−6.0	−5.7	−7.5	−5.8	−5.5	−5.6	−6.8	−7.0	−6.1	−0.06
**D8**	−6.8	−6.6	−7.1	−7.1	−7.4	−6.7	−9.5	−7.3	−6.9	−6.3	−11.3	−8.2	−7.6	1.02
**D9**	−6.6	−6.4	−7.6	−6.4	−7.2	−7.0	−6.8	−6.6	−6.7	−6.6	−6.9	−8.3	−6.9	1.44
**D10**	−7.4	−7.1	−7.3	−7.1	−6.7	−7.9	−9.7	−6.2	−6.6	−6.7	−7.9	−8.7	−7.5	0.29
**D11**	−7.7	−7.3	−6.9	−6.9	−7.0	−7.4	−8.6	−7.4	−7.3	−6.5	−7.5	−7.7	−7.4	1.08
**D12**	−6.7	−7.2	−7.9	−6.8	−7.9	−7.3	−7.5	−7.9	−7.5	−7.0	−11.6	−9.6	−7.9	0.78
**D13**	−7.0	−7.0	−7.3	−7.1	−8.1	−7.3	−7.5	−7.7	−7.2	−6.4	−7.6	−9.2	−7.5	0.71
**D14**	−6.7	−6.5	−6.8	−7.0	−6.9	−7.0	−9.5	−7.4	−7.2	−6.2	−11.8	−7.8	−7.6	0.96
**D15**	−6.8	−6.1	−6.9	−6.5	−6.8	−6.7	−7.9	−7.0	−6.7	−6.4	−6.9	−8.3	−7.0	0.96
**D16**	−5.8	−6.1	−6.9	−5.8	−6.4	−6.0	−6.2	−6.2	−6.6	−6.1	−8.1	−7.2	−6.4	0.25
**D17**	−5.8	−5.3	−5.9	−5.5	−6.3	−5.5	−7.2	−6.0	−5.7	−5.4	−7.0	−7.0	−6.1	0.88
**D18**	−6.4	−6.3	−6.1	−6.2	−7.5	−6.9	−6.9	−6.5	−6.2	−5.9	−6.6	−7.6	−6.6	0.71
**D19**	−6.3	−5.2	−5.7	−5.5	−5.4	−5.5	−6.3	−5.9	−5.1	−4.8	−6.7	−7.0	−5.8	0.68
**D20**	−7.2	−7.2	−7.2	−7.3	−7.5	−7.5	−9.7	−8.2	−7.9	−7.7	−7.6	−8.2	−7.8	0.29
**D21**	−4.2	−4.2	−5.0	−4.3	−4.4	−4.4	−5.4	−4.5	−4.2	−4.3	−5.3	−5.5	−4.6	0.94
**D22**	−6.4	−5.8	−6.3	−6.6	−6.4	−6.2	−6.5	−6.6	−6.3	−5.6	−7.2	−7.1	−6.4	0.62
**D23**	−6.1	−5.8	−6.6	−6.2	−6.5	−6.0	−8.0	−7.4	−6.5	−5.8	−7.0	−6.7	−6.5	1.44
**D24**	−7.2	−7.6	−6.7	−7.5	−8.0	−8.6	−8.8	−8.6	−7.9	−7.0	−7.7	−8.7	−7.9	0.87
**D25**	−6.9	−6.4	−7.1	−6.9	−7.5	−6.7	−9.0	−7.5	−6.7	−5.9	−8.4	−8.7	−7.3	1.54
**D26**	−6.1	−6.3	−6.2	−7.2	−6.7	−7.8	−7.4	−8.2	−7.2	−6.8	−7.1	−7.2	−7.0	0.68
**D27**	−6.3	−6.8	−7.4	−6.5	−6.7	−6.1	−8.4	−6.8	−6.5	−6.5	−8.3	−8.6	−7.1	−0.11
**D28**	−7.9	−7.5	−7.3	−8.8	−7.4	−7.3	−9.5	−7.7	−7.6	−6.8	−12	−9.8	−8.3	0.81

**D1**, Azathioprine (PubChem ID:2265); **D2**, Betamethasone (PubChem ID:9782); **D3**, Clotrimazole (PubChem ID:2812); **D4**, Chlorpheniramine (PubChem ID:2725); **D5**, Clobetasol propionate (PubChem ID:32798); **D6**, Cyclosporine (PubChem ID: 5284373); **D7**, Dapsone (PubChem ID:2955); **D8**, Dexamethasone (PubChem ID:5743); **D9**, Doxycycline (PubChem ID:54671203); **D10**, Apremilast (PubChem ID:5282375); **D11**, Fenretinide (PubChem ID:5288209); **D12**, Fluocinonide (PubChem ID:9642); **D13**, Fluocinolone acetonide (PubChem ID:6215); **D14**, Fluticasone (PubChem ID:5311101); **D15**, Fluticasone propionate (PubChem ID:444036); **D16**, Griseofulvin (PubChem ID:441140); **D17**, Hydroxychloroquine (PubChem ID:3652); **D18**, Isotretinoin (PubChem ID:5282379); **D19**, Levamisole (PubChem ID:26879); **D20**, Methotrexate (PubChem ID:126941); **D21**, Metronidazole (PubChem ID:4173); **D22**, Miconazole (PubChem ID: 4189); **D23**, Mycophenolate mofetil (PubChem ID:5281078); **D24**, Nystatin (PubChem ID:6433272); **D25**, Prednisolone (PubChem ID: 5755); **D26**, Tacrolimus (PubChem ID: 445643); **D27**, Thalidomide (PubChem ID:5426); and **D28**, Triamcinolone acetonide (PubChem ID:6436).

### Virtual screening-cum-molecular docking study

After selection, retrieval, and preparation of 28 repurposed drugs and 12 target protein structures, the PyRx 0.8-AutoDock 4.2 software was used to assess the binding efficacy of individual drugs against individual targets [47–52]. Before molecular docking, we manually analyzed the active site amino acids of each target enzyme, accordingly generated specific grid boxes (see Table S2), and proceeded with the docking study using other default AutoDock parameter setting [47–50]. We generated ten docking poses for individual ligands against individual target proteins, selecting the lower docking pose (kcal/mol) as the most potential interacting pose of each ligand against a respected target. The BIOVIA Discovery Studio Visualizer 2019 (BIOVIA-DSV-2019) software was used to visualize and analyze the protein-ligand interactions (hydrogen bond, van der Wall bond, pi-pi interaction, etc.) of the docking complexes [47, 50].

### Molecular-dynamics simulation and free energy calculations

Based on average docking scores (kcal/mol), two potential docking complexes (GR-**D2**/betamethasone/BETA and GR-**D28**/triamcinolone acetonide/TACA) with apo-protein (GR) were selected for MD simulation at a 200 ns time scale. Docking complexes were subjected to full-atom MD simulations using GROMACS-2020 software with the AMBER99SB-ILDN force field [48–50]. The required ligand topological files for MD simulation were generated by the ACPYPE server. After the addition of solvent molecules and Na⁺ ions for counterbalancing, the energy minimization was performed using 50,000 steepest descent steps for each docking complex [46, 47]. After minimizing the system, the NVT (number of particles, volume, and temperature) and NPT (number of particles, pressure, and temperature) steps were performed for balancing systems, and the final MD production was executed for the protein system at 200 ns with time steps of 2 fs (0.002 ps). The dynamic behavior of both drugs, **D2**/betamethasone/BETA and **D28**/triamcinolone acetonide/TACA, interacting with GR was analyzed using the molecular mechanics/Poisson–Boltzmann surface area (MM/PBSA)-based binding free energy (ΔG_bind_) method from the gmx_MMPBSA suite in the GROMACS platform, followed by the calculation protocol for Gibb’s binding free energy (Δ*G*_bind_) [48–50, 53].

### Frontier molecular orbital analyses

The reactivity, stability, and molecular interactions of two potential steroidal (**D28**/triamcinolone acetonide/TACA and **D2**/betamethasone/BETA) and two non-steroidal (**D10**/apremilast/APR and **D20**/methotrexate/MTX) drugs were analyzed through FMO analyses by calculating the highest occupied molecular orbital (HOMO), the lowest occupied molecular orbital (LUMO), and the energy gap (ΔE_H_-_L_) [46, 48]. We conducted electronic structure calculations using the restricted Hartree-Fock (RHF) model, performing a single-point energy calculation after optimizing energy with the MMFF94 force field, and then visualized the HOMO-LUMO energy plots using Avogadro-ORCA 1.2.0 software [46, 48].

### Drug-ability prediction and SAR analyses

Using advanced computational tools, drug-likeness (DL) scores are also possible for any structure comparison with training set datasets. Therefore, we have predicted individual DL-scores for all drug candidates using the Molsoft (http://molsoft.com/mprop/) tool. We predicted the DL-score, primarily to cross-check and measure the reliability of the computational tool as well as to select the potential one from among them [46, 48]. On the other hand, we conducted structural activity relationship (SAR) analyses to investigate the potency of drug candidates in relation to their chemical structure [49, 50]. Based on their potency and similar cores, we selected nine steroidal candidates from our list. For SAR analyses, we have represented all chemical structures using ChemDraw 18.0 software and analyzed their drug chemistry by comparing the functional attachments [46, 48].

## Results

### Network pharmacology to explore disease networks and putative targets of OLP

**Fig. 1:**
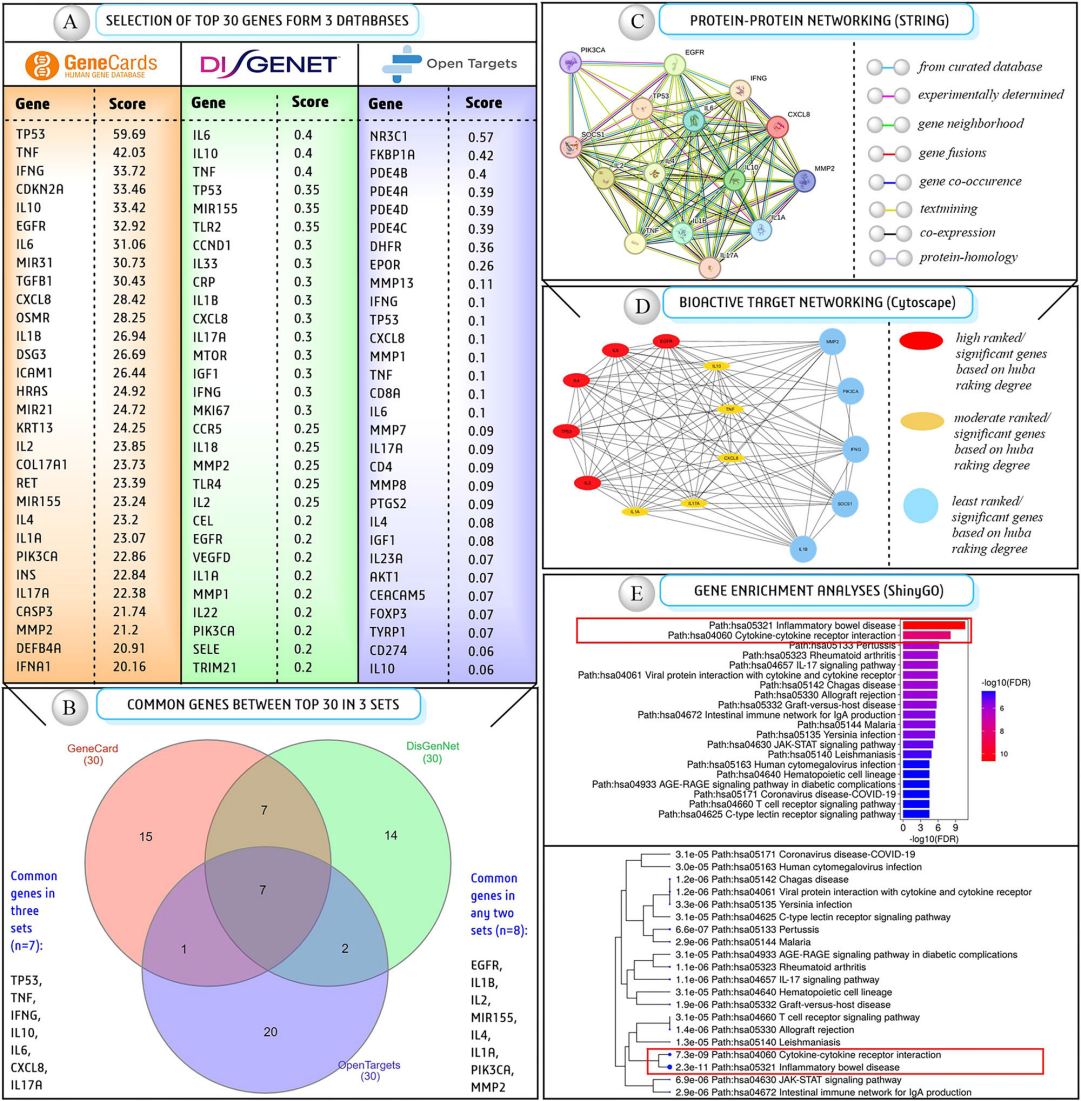
The process of selecting the most putative target genes associated with OLP is followed by systematic system biology and network pharmacology. **A** Top thirty selected candidate genes based on global score from three different curated databases for further investigation; **B** Common genes among three data sets and presented in a minimum of two data sets presented through a vein diagram; **C** Protein–protein networking analyses to explore the disease biology through the STRING database; **D** Selection of the most bioactive candidate genes through Cytoscape; **E** Gene enrichment and KEGG pathway analyses to select the final set of putative genes to be used as targets in further computational investigation.

To identify most putative target genes, network pharmacology is an ideal platform, where we initially chose a vast array of genes from three curated databases, including 366 genes from DisGeNET, 594 genes from GeneCards, and 596 genes from Open Targets. Further, we only selected the top 30 relevant genes based on their global score ranking, which represents their high relevance to the disease network (Fig. 1A). Among all three sets, we found that seven genes (TP53, TNFα1, IFNγ, IL10, IL6, CXCL8/IL8, and IL17α) are common. Additionally, eight genes (EGFR, IL1B, IL2, MIR155, IL4, IL1α, PIK3CA, and MMP2) are identical in any two data sets based on manual analyses; these findings are presented in a Venn diagram **(**Fig. 1B). Furthermore, the PPI network demonstrated a robust interaction among the genes, as all were presented in a single interactive network (Fig. 1C). Taking this STRING dataset, we used Cytoscape to select bioactive targets and found EGFR, IL6, IL4, TP53, and IL6 were the top candidates, and IL1α, IL17α, CXCL8, TNFα1, and IL10 were the second-most relevant candidates based on the huba-ranking degree (Fig. 1D). In addition, the gene or fold enrichment report revealed that inflammatory bowel diseases and cytokine-cytokine receptor interaction are two of the most relevant biological processes/pathways associated with OLP and presented two branches of a single node in the phylogenetic tree (Fig. 1E). Finally, after KEGG pathway analyses, we selected IL1α, IL2, IL4, IL6, IL10, and IL17α as the most putative target genes for our study (Fig. S1). Simultaneously, OLP is an autoimmune skin disorder that lacks extensive biological information, and continuous experimental and clinical investigations are going to explore the disease’s network of several target-specific genes using patient-derived RNA sequencing and genome sequencing data. As a result, to explore more relevant and reliable target-specific therapeutic information, we selected another set of candidate genes, TNFα1, IFNγ3, NFKB, HSP70, GR, and TLR7, to form recent clinical reports to explore the potency in an unbiased approach.

### Virtual screening-cum-molecular docking study

**Fig. 2:**
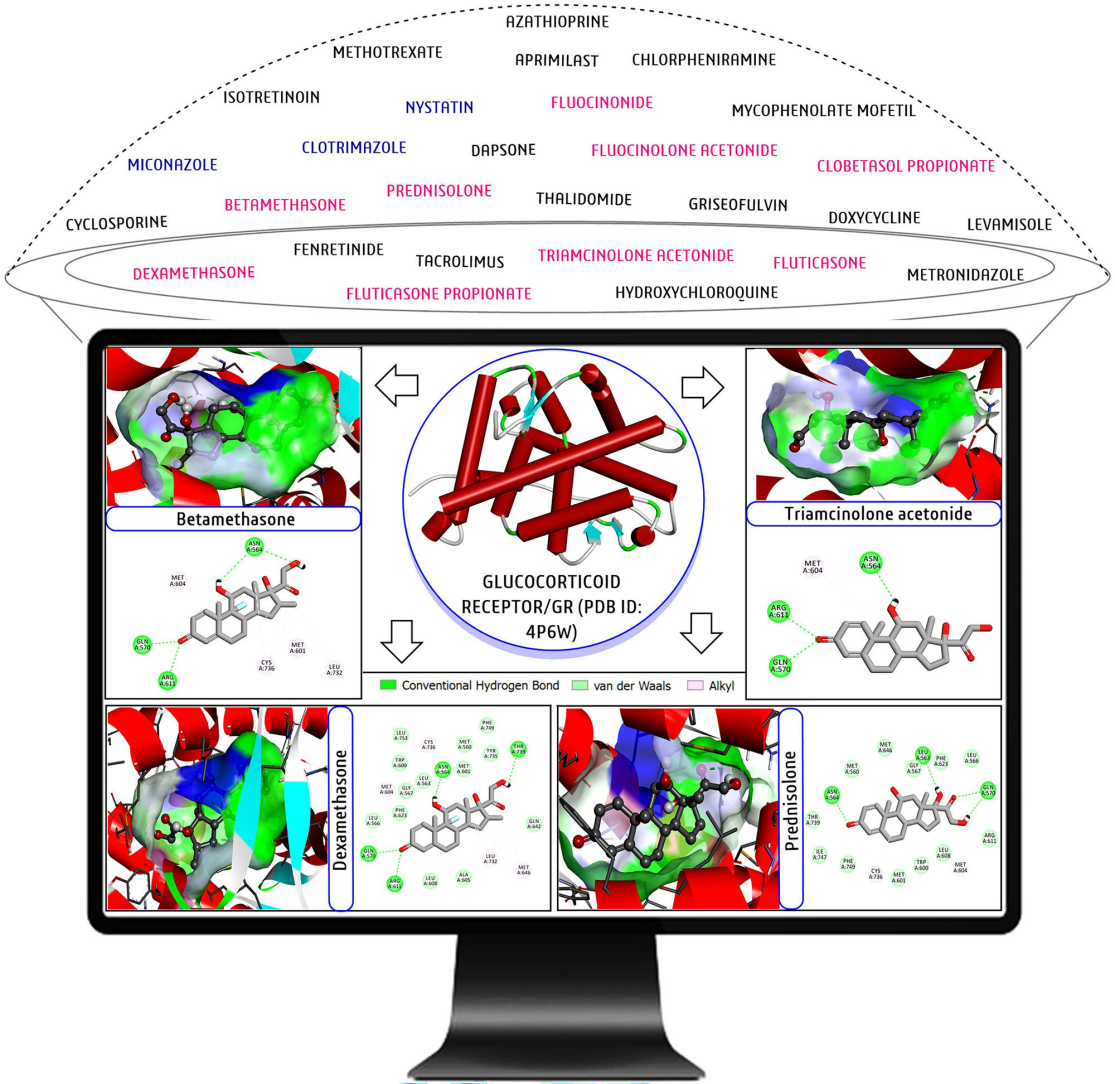
A schematic representation of selected drug candidates (D2/Betamethasone/BETA, D28/triamcinolone acetonide/TACA; D8/dexamethasone/DEX, and D25/prednisolone/POM) for the present study to explore the target-specific efficacy with glucocorticoid receptors (GR). The BIO-VIA Discovery Studio Visualizer and ChemDraw 2020 software were used to graphically present the protein-ligand interaction of four potential steroidal drugs against GR.

Individual docking scores (kcal/mol) of all 28 repurposed drugs against 12 targets are presented in Table 1 (see the footnote in Table 1 for detailed information on the 28 drugs presented as **D1**-**D28**). Noted that the lower in binding energy (higher in negative value) represents the higher the efficiency according to AutoDock software [46, 48]. Accordingly, we recorded drug efficacy in the form of docking scores and found it within a range of −4 to −13 kcal/mol, whereas the average docking score of each individual drug against 12 targets was between −6 and −10 kcal/mol (Table 1). According to recorded docking scores, **D28** (triamcinolone acetonide/TACA) showed a strong binding efficacy against GR (−12 kcal/mol), and **D21** (Metronidazole/MTZ) demonstrated poor binding efficacy (−4.2 kcal/mol) against IL1α, IL2, and NF_K_B (Table 1). Based on 28 drugs against individual targets, the **D28** (triamcinolone acetonide/TACA) against IL1α (−7.9), **D2** (betamethasone/BETA) against IL2 (−9.9), **D12** (fluocinonide/FLOC) against IL4 (−7.9), **D28** (triamcinolone acetonide/TACA) against IL6 (−8.8), **D13** (fluocinolone acetonide/FCLA) against IL10 (−8.1), **D24** (nystatin/NYT) against IL17α (−8.6), **D2** (betamethasone/BETA) against TNFα1 (−9.9), **D24** (nystatin/NYT) against IFNγ3 (8.6), **D20** (methotrexate/MTX) and **D24** (nystatin/NYT) against NF_K_B (−7.9), **D20** (methotrexate/MTX) against HSP70 (−7.7), **D28** (triamcinolone acetonide/TACA) against GR (−12), and **D28** (triamcinolone acetonide/TACA) against TLR7 (−9.8), recorded as most potential (Table 1). Similarly, based on each drug’s average docking score against all 12 targets, **D28/**triamcinolone acetonide/TACA (−8.3), **D2/**betamethasone/BETA (−8.2), **D12/**fluocinonide/FLOC and **D24/**nystatin/NYT (−7.9), **D20/**methotrexate/MTX (−7.8), **D8/**dexamethasone/DEX and **D14/**fluticasone/FP (−7.6), **D10/**apremilast/APR and **D13/**fluocinolone acetonide/FCLA (−7.5), and **D11/**fenretinide/4−HPR (−7.4) are the top ten potential drug candidates. We clearly observe that each drug exhibits varying interaction efficacy against each target. Since all drugs function through repurposing without any specific mode of action, the target-specific interaction aids in understanding potential mechanisms for target therapy. Furthermore, using the BIOVIA-DSV-2019 software, we visualized the protein-ligand interactions (3D and 2D) of four selective potential drugs against GR (Fig. 2). The interaction analyses revealed the presence of 3–4 hydrogen bond interactions and pi-alkyl van der Wall interactions in each docking complex, which helps to formation of strong binding efficacy with GR (Fig. 2). In overall records and potency, we selected GR- **D28**/triamcinolone acetonide/TACA and GR-**D2**/betamethasone/BETA for MD-simulations.

### Molecular-dynamics simulation and free energy calculations

**Fig. 3:**
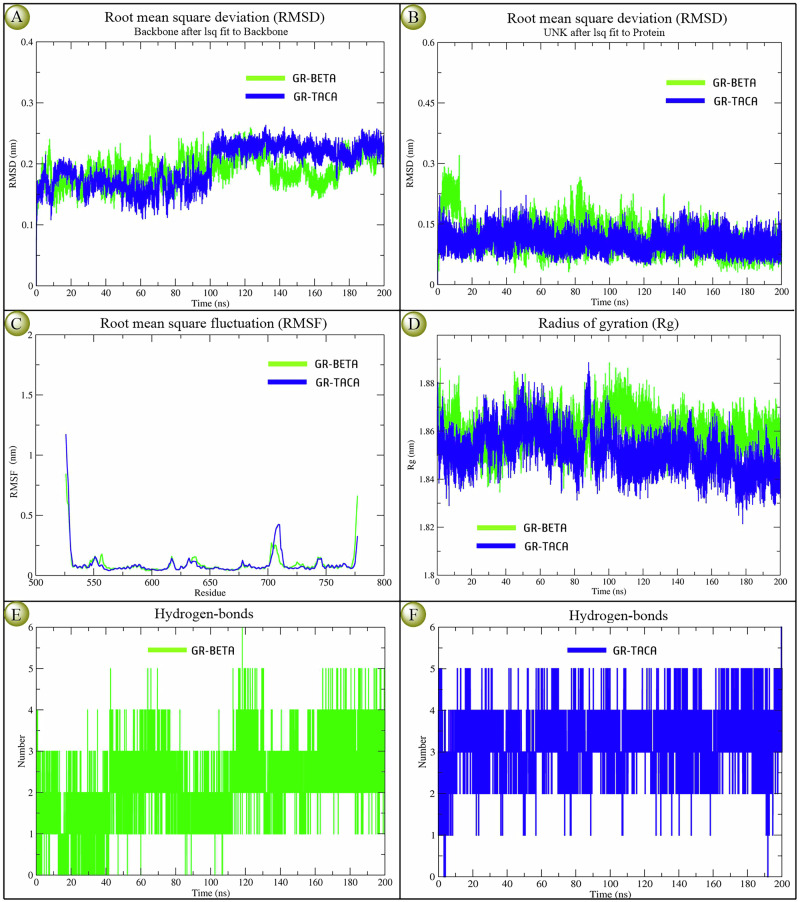
Conformational stability in the form of RMSD, RMSF, and Rg-score plots of apo-protein (GR), GR-**D28**/triamcinolone acetonide/TACA and GR-**D2**/betamethasone/BETA, docking complexes within 200 ns. **A** overlaid RMSD-plots of backbone protein; **B** overlaid RMSD-plots of ligand; **C** overlaid RMSF-plots; **D** overlaid Rg-plots; **E** H-bond interaction plot of the GR-**D2**/betamethasone/BETA; and **F** H-bond interaction plot of the GR- **D28**/triamcinolone acetonide/TACA.

Based on generated RMSD, RMSF, Rg, and H-bond interaction plots (Fig. 3A–F), we have observed the molecular stability and protein kinetic behaviors of GR-**D28**/triamcinolone acetonide/TACA and GR-**D2**/betamethasone/BETA docking complexes at 200 ns (Fig. 3), as well as apo-protein, GR (Fig. S2). According to the RMSD plots, both complexes exhibited similar types of deviation up to 100 ns, within a range of 0.25 to 0.1 nm. However, after that, **D28**/triamcinolone acetonide/TACA demonstrated higher stability within a range of 0.2–0.25 nm, compared to **D2**/betamethasone/BETA, within a deviation range of 0.1 to 0.2 nm up to 200 ns (Fig. 3A). The depicted overlaid ligand RMSD plots also indicated that the **D28**/triamcinolone acetonide/TACA complex was comparatively more stable than **D2**/betamethasone/BETA (Fig. 3B). Similarly, the RMSF and Rg- plots showed that both complexes had similar types of deviations, with only a very small deviation observed between them (Fig. 3C, D). The number of H-bond interactions also determines the stability of protein-ligand stability, where both docking complexes exhibited five maximum H-bond interactions within 200 ns. However, the GR-**D28**/triamcinolone acetonide/TACA complex demonstrated a maximum of two to five H-bond interactions, whereas the GR-**D2**/betamethasone/BETA complex only displayed one to three H-bond interactions (Fig. 3E, F). Overall, both steroidal drugs showed strong and similar types of stability with GR, especially GR-**D28**/triamcinolone acetonide/TACA, which was comparatively more stable than GR-**D2**/betamethasone/BETA from a minute observation.

**Fig. 4:**
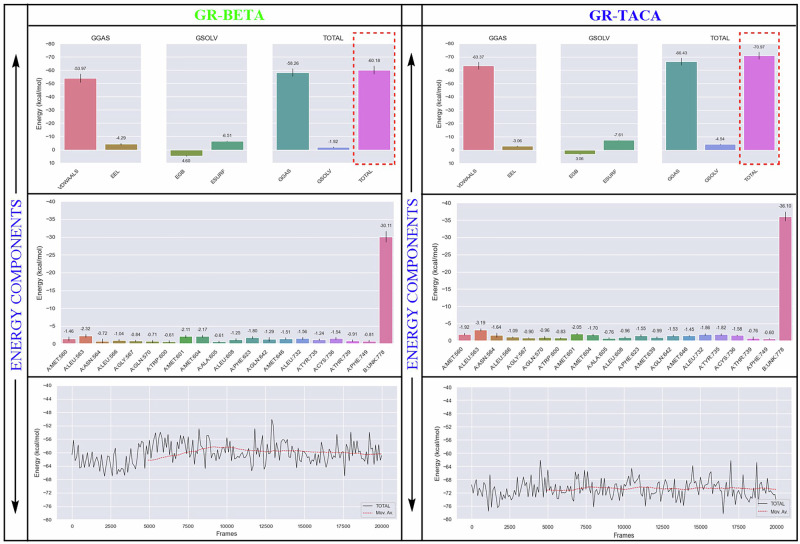
The Gibbs free energy (Δ*G*_bind_) calculation using the MM/PBSA method. Based on total binding energy reports, GR-**D28**/triamcinolone acetonide/TACA (70.97 kcal/mol) displayed comparatively higher stability than GR-**D2**/betamethasone/BETA (−60.18 kcal/mol) with the least free energy.

Further, the free energy (MM/PBSA) was calculated for both docking complexes and it was found that GR-**D28**/triamcinolone acetonide/TACA had a higher binding energy of (−70.97 kcal/mol), than GR-**D2**/betamethasone/BETA (−60.18 kcal/mol), as similar trends to MD simulation (Fig. 4). Using the Gibbs free energy equation (Δ*G*_bind_), we calculated each energy component ultimately determining the total binding free energy of both docking complexes (Fig. 4). Additionally, we observed that both complexes share nineteen common residues, including MET560, LEU563, ASN564, LEU566, GLY567, GLN570, TRP600, MET601, MET604, ALA605, LEU608, PHE623, GLN642, MET646, LEU732, TYR735, CYS736, THR739, and PHE749. These residues contribute to the high levels of binding energy (Fig. 4). The movable energy line, which spans from 1 to 200 ns, shows that GR-**D28**/triamcinolone acetonide/TACA has a straighter line than GR-**D2**/betamethasone/BETA does. Among the two ligand molecules, TACA exhibited the stronger binding affinity (−36.10 kcal/mol) compared with BETA (−30.11 kcal/mol), as presented in Fig. 4. Overall, based on MD and MMPBSAS, it showed that **D28**/triamcinolone acetonide/TACA had comparatively more potential and was more stable with GR than **D2**/betamethasone/BETA.

### Frontier molecular orbital analyses

**Fig. 5:**
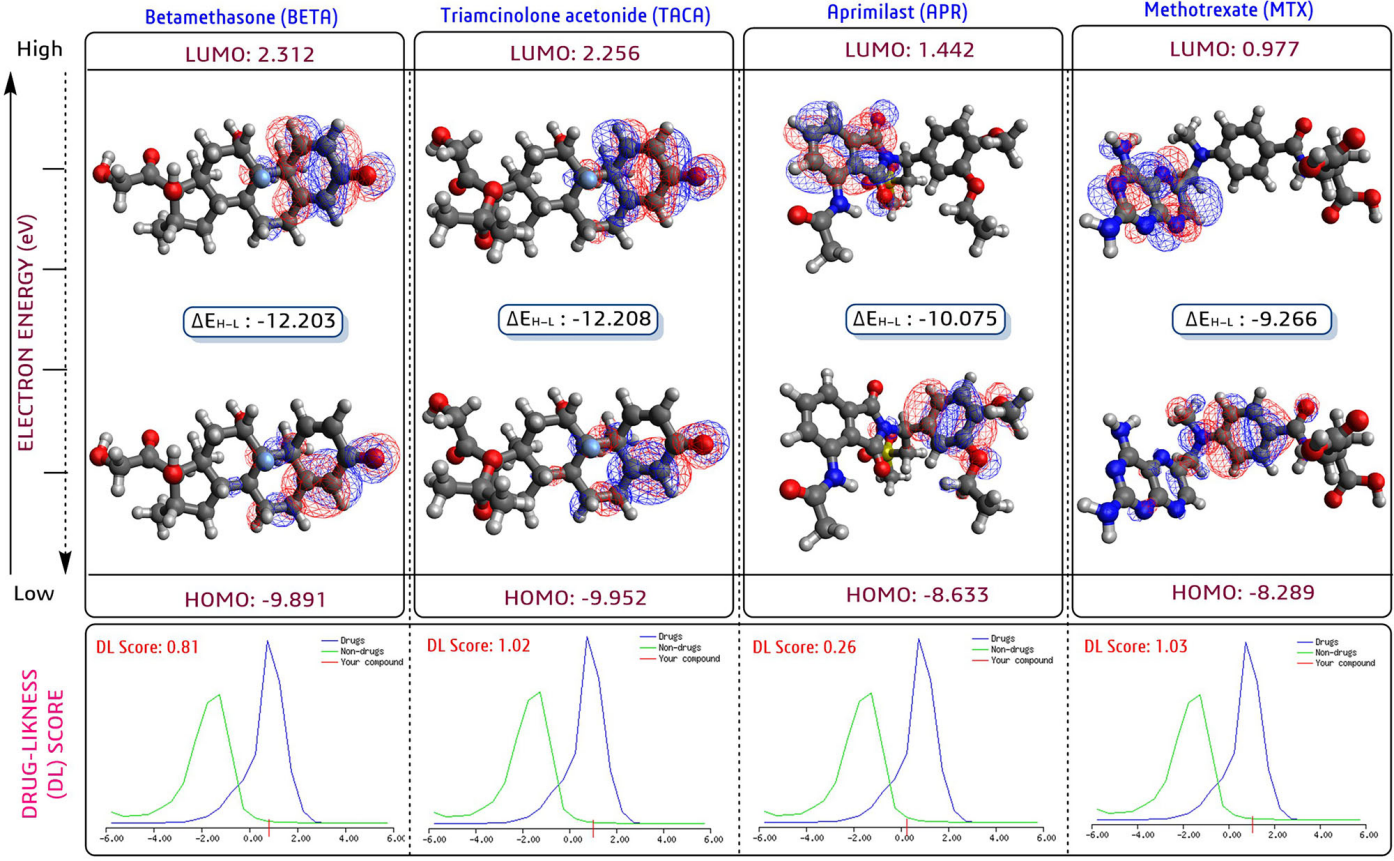
The FMO analyses in the form of HOMO, LUMO and their energy gap (Δ*E*_H-L_ in eV) of two steroidal (**D2**/betamethasone/BETA and **D28**/triamcinolone acetonide/TACA) and two non-steroidal (**D10**/apremilast/APR and **D20**/methotrexate/MTX) drugs to analyze flexibility and reactiveness with target protein in an orbital theory perspective. Additionally, the Molsoft tool predicts the drug likeness score in a graphical form for the four drugs mentioned above.

The FMO analyses in the form of HOMO (nucleophilic), LUMO (electrophilic), and their respective energy gap (Δ*E*_H-L_ in eV) surface molecular energy plots were depicted in Fig. 5. Briefly, the calculated ΔE_H-L_ of **D2**/betamethasone/BETA and **D28**/triamcinolone acetonide/TACA was −12.203 and −12.208 eV, whereas **D10**/apremilast/APR and **D20**/mthotrexate/MTX were −10.075 and −9.266 eV, which indicated that **D2**/betamethasone/BETA and **D28**/triamcinolone acetonide/TACA were more flexible and reactive than **D10**/apremilast/APR and **D20**/methotrexate/MTX (Fig. 5). Higher HOMO, LUMO, and ΔE_H-L_ indicate **D2**/betamethasone/BETA and **D28**/triamcinolone acetonide/TACA are in a transition state or excited state, resulting in a higher reactivity with the target GR. According to analyses of chemical structure and surface energy, cyclohexane rings (A and B) are very reactive in both steroidal drugs, according to HOMO and LUMO energy plots (highlighted in spherical and oval-shaped mesh cells). On the other hand, the 1-(3-ethoxy-4-methoxyphenyl) group displayed the HOMO surface, while the isoindole 1,3-dione moiety displayed the LUMO surface in **D10**/apremilast/APR. In **D20**/methotrexate/MTX, the 4-aminobenzamide group occupied the HOMO surface, while the pteridin moiety occupied the LUMO surface (Fig. 5). Overall, HOMO-LUMO expresses the nature of chemical structure (flexibility and rigidity), which we have correlated with binding affinity [46, 48].

### Drug-ability and SAR analyses

The Molsoft tool predicted the drug-ability profiles of all drug candidates (Table 1). Apart from **D1**/azathioprine/AZA, **D3**/clotrimazole/CLT, **D6**/cyclosporine/CyA, **D7**/dapsone/DDS, and **D27**/Thalidomide/THD, the rest of the drugs showed a higher drug-ability score. Among all, **D11**/fenretinide/4-HPR, **D14**/fluticasone/FP, **D17**/hydroxychloroquine/HCQ, **D24**/nystatin/NYT, and **D28/**triamcinolone acetonide/TACA had higher drug-likeness scores >1 (0.96, 0.88, 0.87, and 0.81), while **D2**/betamethasone/BETA, **D4**/chlorpheniramine/CPM, **D9**/doxycycline/DOX, **D23**/mycophenolate mofetil/MMF, and **D25/**prednisolone/POM had <1 (1.02, 1.97, 1.44, 1.44, and 1.54) (Fig. 5). Technically, the Molsoft tool presents both drug and non-drug information; upon entering a chemical, the engine searches, compares the structure, and subsequently provides a statistical score. According to the Molsoft tool, any candidates fall within a range of 0 to 2, indicating their suitability and chance of higher experimental success. All selected drugs are already available on the market for various therapeutic uses, so predicting the score may not be necessary. However, this tool is particularly helpful in selecting the most potential among a series of drugs, a process that is not possible in in vitro studies. Therefore, most academic, drug-developer, and pharmaceutical researchers use this tool to select ideal candidates for further progress in synthesis and experimental work, with the aim of achieving a higher rate of success [46, 48]. Overall, drug-likeness is the sum of a candidate’s physiochemical, pharmacokinetic, and toxicity profiles based on their chemical structure [47–50].

**Fig. 6:**
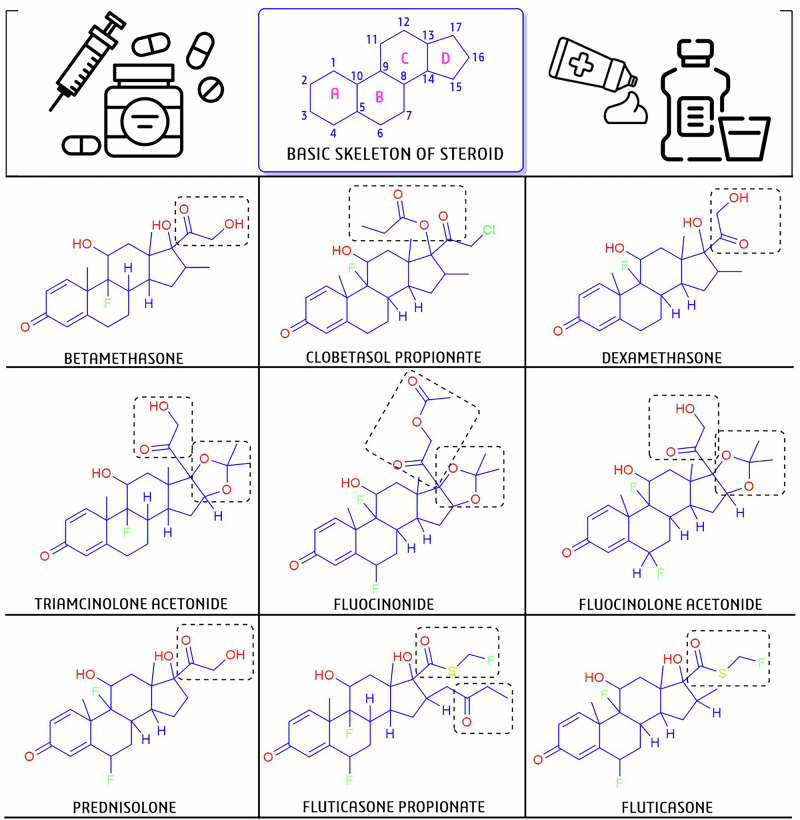
Structural activity relationship (SAR) analysis of nine steroidal drugs among 28 selected lists. We used ChemDraw 2020 software to make the chemical structure during SAR analyses, where the various functional attachments were highlighted in dotted boxes.

The SAR, a pioneer concept in medicinal chemistry and drug discovery, helps to explore the drug chemistry or biological activity related to chemical structure [54, 55]. As per the hypothesis, we have exclusively analyzed the SAR of nine steroid drugs with docking and drug-likeness scores (Fig. 6). Generally, four rings fuse together to form a single steroid moiety system, as presented in Fig. 6 [55, 56]. Among the nine steroid classes of drugs containing common pregnane nuclei and substituted fluorine with different functionalities in individual structures, the illustrated drugs are hydroxymethyl acetone (COCH_2_OH) in **D2**/betamethasone/BETA, **D8**/dexamethasone/DEX, **D28**/triamcinolone acetonide/TACA, **D13**/fluocinolone acetonide/FCLA, and **D25**/prednisolone/POM. Ester of propionic acid (CH_3_CH_2_COO^-^) in **D5**/clobetasol propionate/CP; acetic hypofluorous thioanhydride (C_2_H_3_FOS) and ethyl methyl ketone (-C(=O)CH_2_CH_3_) in **D15**/fluticasone propionate/FP; and acetonide-2,2-dimethyl-1,3-dioxolane (C_5_H_10_O_2_) in **D28**/triamcinolone acetonide/TACA, **D12**/fluocinonide/FLOC, and **D13**/fluocinolone acetonide/FCLC, respectively (Fig. 6). From a SAR perspective, the varying functional attachment of the core nuclei provides different characteristics, thereby aiding or encouraging the development of additional therapeutic candidates. In this regard, a number of plant-derived steroids with a similar core structure have shown promise for anti-inflammation, antioxidants, and immune modulation potency, and they could be ideal alternative candidates to use for OLP management [57–59].

## Discussion

Currently, researchers have identified over 100 types of autoimmune diseases, including OLP, but no recommended therapy exists for their control and management [9–11]. In the case of OLP, the pathophysiology has not been thoroughly studied; as a result, physicians have recommended various repurposed drugs to manage the symptoms, reduce inflammation, and protect mucosal damage. Physicians prefer and recommend steroidal therapy due to its faster reduction of skin lesions and pain compared to other therapies, but the effectivity was widely distinct in each individual. In addition, steroidal therapy is associated with several adverse effects, including mucosal thinning, candidiasis, adrenal suppression from corticosteroids, and burning sensations, which increase the risk of malignancy from calcineurin inhibitors as per the reports [16, 17, 23, 27]. Similarly, retinoid therapy can cause mucosal irritation, a high risk of acitretin, liver toxicity, and teratogenicity, while immunosuppressant therapy comes with side effects and a high treatment cost [30, 31]. Till date, most therapies are not curative, focusing on symptom management rather than addressing the underlying autoimmune cause. Therefore, target therapy with long-term management strategies is often required. In comparison to present computational study with existing clinical data, BETA and TACA are widely used and effective drugs at present for OLP. From both potency and adverse effect points of view, TACA is widely recommended for potency and recorded a lower incidence of systemic adverse effects [60, 61]. In addition, TACA shows a favorable safety profile from long-term clinical management of oral lesions even at lower concentrations [60, 61]. Although BETA also exhibits potency against OLP, its systemic absorption and prolonged use are associated with several adverse effects, such as mucosal thinning, secondary infections, etc [62, 63]. By integrating these clinical reports with our present computational findings, we suggest that TACA may offer an improved therapeutic margin for OLP management.

In this context, the current study implements advanced network pharmacology to systematically explore disease biology and recognize ideal targets through network pharmacology for OLP [64–66]. It provided comprehensive insights into gene function and drug-target interactions, allowing us to systematically analyze the complex biological networks as well as the most potential repurposed drugs for OLP [44, 67]. Additionally, we incorporated several targets from recent literature to evaluate the therapeutic index of our twenty-eight repurposed candidates in a methodical and reliable approach. Although, a huge amount of genomic and proteomic data has been continuously available publicly, which data may be significantly helpful to explore the disease biology and personalized medicine to treat autoimmune diseases like OLP [32–34]. Recently, there have been several omics studies underway to reveal the pathogenesis of OLP through RNA sequencing, transcriptomic profiles, and next-generation sequencing [66, 68, 69]. Wang and Co. conducted a study that examined the transcriptomic profiles of six normal and six OLP-infected oral tissues and found five RYK (receptor-like tyrosine kinase), SLC8A1 (solute carrier family 8 member A1), WDR7 (WD repeat domain 7), MAP3K5 (mitogen-activated protein kinase 5), and GPBP1 (GC-rich promoter binding protein 1) out of 153 candidate genes to be highly intramodular and connected and could be used as new targets for diagnosis and treatment [69]. Furthermore, a new study by Vo and others found that IL36γ, also known as interleukin-1 family member 9 (IL1F9), was a key regulator in wound healing and antimicrobial defense and suggested it may be possible to treat using transcriptome profiles of OLP tissues [70]. Overall, the network pharmacology and CADD platform provides a cost-effective platform for exploring the ideal target for the development of a newer drug in a target-specific manner for the developer and selection of alternative therapeutic candidates [32–34, 46–50].

Using advanced bioinformatics tools, researchers are also trying to locate promising natural candidates against various emerging and neglected diseases, including OLP [28, 44, 67, 70–73]. Traditionally, several anti-inflammatory and antioxidant natural herbal regimens, such as *Curcuma longa*, *Aloe vera, Chamaemelum nobile, Camellia sinensis, Portulaca oleracea, Paeonia lactiflora*, *Glycyrrhiza glabra*, etc., crude extracts, and their derived phytoconstituents, such as curcumin, quercetin, catechin, β-carotene, etc., are considered potential alternative and complementary therapies for OLP, as most of the natural regimens are under clinical trial investigation [71,74]. Despite the lack of recommended FDA-approved target therapies and gold standard in vitro models, the CADD platform serves as an ideal tool to evaluate the potency of desired candidates in a target-specific manner [46–50]. Currently, the CADD platform is widely used to explore the potency, binding efficiency, drug chemistry, and pharmacological insights of any set of desired candidates, and accordingly, the present study locates an effective drug candidate from an existing repurposing list for the management of OLP.

## Conclusion

Currently, FDA-approved targeted therapies are not available for OLP; as a result, physicians have repurposed a number of drugs to control OLP lesions and pain. Clinical reports indicate that, among all classes, steroidal therapy stands out as a promising treatment with a relatively high efficacy, yet it often results in additional health complications and adverse effects for patients. Therefore, the advanced CADD platform is able to explore the disease network, identify putative targets, and locate potential drug candidates through molecular docking, MD simulation, free energy calculation, FMO, SAR, and drug-ability analyses. Currently, network pharmacology is one cost-effective platform in CADD that is used to identify the potential molecular target for future drug discovery, where molecular docking simulation is widely used in the academic and pharmaceutical sectors to assess the target-specific efficacy of any desired candidates. In conclusion, we found that among the twenty-eight repurposed drug lists, injectable **D28**/triamcinolone acetonide/TACA could be a better option for OLP management.

## Supplementary information

SUPPLEMENTARY FILE

## Data Availability

This published article and its supplementary information file contain data generated and analyzed to support this study. Data will be made available on request.
