# Supplementary material for: Target-specific therapeutic assessment of repurposed drug candidates for oral lichen planus: a network pharmacology-molecular dynamics simulation guided investigation
Source: BDJ Open. 2025 Dec 19;11:99. doi: 10.1038/s41405-025-00384-y (PMC12717163; doi:10.1038/s41405-025-00384-y)
Supplement: Supplementary file 1 — SUPPLEMENTARY FILE [file 41405_2025_384_MOESM1_ESM.docx]

**SUPPLEMENTARY DATA**

**Target-specific therapeutic assessment of repurposed drug-candidates for Oral lichen planus: A network pharmacology-molecular dynamics simulation guided investigation**

Alaka Sahoo ^a,b,c^, Shasank Sekhar Swain ^c,d^, Satya Ranjan Singh ^e^, Atala Bihari Jena ^f^, Sudhir Kumar Paidesetty ^b^, Asim K. Duttaroy ^g^_,_ Maitreyee Panda ^a,*^

**Table S1.** Currently prescribed mainstream drugs for OLP treatment with bioactivities, mechanisms, and molecular formulae and PubChem reference IDs other relevant information.

| **Sl. No** | **Drug name**  **(drug/ chemical class)** | **Reported/ existing activities** | **Mechanism of action/ human targets** | **PubChem ID**  **(MW g/mol.)** | **Reference** |
| --- | --- | --- | --- | --- | --- |
|  | Azathioprine ** (thiopurine/ imidazole thioether) | Anti-inflammation, vesiculobullous, autoimmune, and connective tissue disorders, etc. | Purine synthesis, inhibits B and T cells,ras-related C3 botulinum toxin substrate-1 | 2265 (C_9_H_7_N_7_O_2_S) | Didona et al., 2022; Verma et al., 2001 |
|  | Betamethasone #* (hydroxy fluorinated steroid) | Anti-inflammation, immunosuppressives, skin infection, etc. | NF-Kappa B, phospholipase A2, glucocorticoid receptor | 9782 (C_22_H_29_FO_5_) | Ezzatt and Helmy, 2019; Gamal Alsakaan et al., 2022; Samimi et al., 2020 |
|  | Clotrimazole # (triphenyl imidazole derivate/ ergosterol) | Anti-fungal, sickle cell disease, malaria, cancer, etc. | Ergosterol, lanosterol 14-demethylase, Ca2+‐ATPase | 2812 (C_22_H_17_ClN_2_) | Marable et al., 2016 |
|  | Chlorpheniramine * (amine/ pheniramine derivative) | Allergic, rhinitis, cold fever, urticaria, allergy, asthma, etc. | Histamine H1 receptor, sodium-dependent serotonin transporter | 2725 (C_16_H_19_ClN_2_) | Vaishnavi and Santhosh, 2022 |
|  | Clobetasol propionate # (prednisolone derivative) | Anti-inflammatory, plaque psoriasis, pruritic, etc. | Glucocorticoid receptor, NF-Kappa B, phosphor-lipase A2, interleukin-10 | 32798 (C_25_H_32_ClFO_5_) | Mamadapur et al., 2022; Kumar et al., 2022; Arduino et al., 2018; Brennan et al., 2022 |
|  | Cyclosporine **  (cyclic non-ribosomal peptide) | Immunosuppressants, arthritis, ulcerative colitis, uveitis, etc. | Cyclophilin-1 receptor, nuclear factor of activated T cells (NF-AT) | 5284373 (C_62_H_111_N_11_O_12_) | Monshi et al., 2021; Georgaki et al., 2022 |
|  | Dapsone *#  (sulfonamide class drug) | Immunosuppressive, acne vulgaris, leprosy, microbial infection, skin disorder, etc. | Dihydropteroate synthetase-1 and -2 (folic acid synthesis) | 2955 (C_12_H_12_N_2_O_2_S) | Singh et al., 2017; Verma and Pandhi, 2015 |
|  | Dexamethasone ***  (glucocorticoid / steroid) | Allergic, rheumatic, SARS-CoV-2, dermatologic, respiratory disorders, etc. | Glucocorticoid receptor, phospholipase A2, NF-Kappa B, interleukin-10 | 5743 (C_22_H_29_FO_5_) | Georgaki et al., 2022; Villa et al., 2020; Zhang et al., 2022; Hambly et al., 2017 |
|  | Doxycycline **  (semisynthetic tetracyclic) | Antibacterial, periodontitis, acne, SARS-CoV-2, respiratory/ pneumonia, etc. | Bacterial 30S and 50S subunits, aminoacyl-tRNA **(lead to protein synthesis)** | 54671203 (C_22_H_24_N_2_O_8_) | NCT00484250; Arash et al., 2008; Piacentini et al., 2019 |
|  | Apremilast | Anti-inflammatory, | Inhibits the Phosphodiesterase-4 (PDE-4), cyclic adenosine monophosphate (cAMP), tumor necrosis factor-alpha (TNF-α) | 11561674  (C_22_H_24_N_2_O_7_S) | Perschy et al., 2022; Bettencourt et al., 2016; Kim-Lim et al., 2023; AbuHilal et al., 2016 |
|  | Fenretinide *  (phenylretinamide retinoid derivative) | Macular degeneration, anti-neoplastic (breast cancer), chemo-preventive, etc. | Inhibits the growth of human cancer cell lines via retinoid receptor | 5288209 (C_26_H_33_NO_2_) | Rotaru et al., 2020; Petruzzi et al., 2013 |
|  | Fluocinonide # (corticosteroid/ steroid) | Antiinflammatory, antipruritic, skin disorder like eczema, etc. | Glucocorticoid receptor, phospholipase A2, cyclo-oxygenase -1 and -2 | 9642 (C_26_H_32_F_2_O_7_) | Davari et al., 2014 |
|  | **Fluocinolone acetonide** # (corticosteroid/ steroid) | Antiinflammatory, diabetic edema, vasoconstrictive, pruritic, skin disorders, etc. | Glucocorticoid receptor, phospholipase 2, annexin A1-A5 (inducer) | 6215 (C_24_H_30_F_2_O_6_) | Thongprasom, 2017; Buajeeb et al., 2000; Saengprasittichok et al., 2022 |
|  | Fluticasone #$  (glucocorticoid/ steroid) | Antiinflammatory, asthma, allergic rhinitis, pruritic dermatoses, etc. | Glucocorticoid receptor, NF-Kappa B, cytosolic phospholipase A2 | 5311101 (C_22_H_27_F_3_O_4_S) | Ynson et al., 2013 |
|  | Fluticasone propionate #$ (glucocorticoid/ steroid) | Asthma, inflammatory pruritic dermatoses, nonallergic rhinitis, etc. | Glucocorticoid receptor, cytosolic phospholipase A2,cyclooxygenase 2 | 444036 (C_25_H_31_F_3_O_5_S) | Hegarty et al., 2002; Ynson et al., 2013; Donnellan et al., 2011 |
|  | Griseofulvin *#  (benzofurans derivative) | Fungal associated skin, hair, nails infections (tinea capitis, pedis, corporis, cruris), ringworm, etc. | Inhibit fungal cell mitosis, nuclear acid synthesis via α and β-tubulin, keratin type-I cytoskeletal-12 | 441140 (C_17_H_17_ClO_6_) | Matthews and Scully, 1992; Naylor, 1990 |
|  | Hydroxychloroquine* (chloroquine/ quinine derivative) | Immunosuppressive, lupus erythematosus, arthritis, malaria, SARS-CoV-2, etc. | Toll-like receptor 7 and 9, angiotensin-converting enzyme-2 | 3652 (C_18_H_26_ClN_3_O) | Xie et al., 2023; Yeshurun et al., 2019; Raj et al., 2021 |
|  | Isotretinoin *  (retinoic acid or retinoid derivative) | Antiinflammatory, skin diseases, neoplastic, recalcitrant acne, etc. | Retinoic acid receptor-α and -γ | 5282379 (C_20_H_28_O_2_) | Petruzzi et al., 2013 |
|  | Levamisole *  (synthetic imidazothiazole derivative) | Anthelminthic, rheumatoid arthritis, immunomodulator, parasitic, bacterial, viral infection, etc. | Neuronal acetylcholine receptor subunit α-3, alkaline phosphatase receptors (unc-29, 38, 63) | 26879 (C_11_H_12_N_2_S) | Chiang, 2012; Lu et al., 2019; Won et al., 2009 |
|  | Methotrexate *  (Folic acid or aminopterin derivative) | Antiinflammatory, juvenile and rheumatoid arthritis, psoriasis, leukaemia with a variety of cancers, etc. | Dihydrofolate reductase, thymidylate synthase, bifunctional purine synthesis protein | 126941 (C_20_H_22_N_8_O_5_) | Gamal Alsakaan et al., 2022; Lajevardi et al., 2016; Goel and Khurana, 2021; Chauhan et al., 2018 |
|  | Metronidazole ***  (nitroimidazole class of antibiotic) | Antiinflammatory, skin disorder, gastrointestinal infections, antibacterial, anti-protozoal, antiparasitic, etc. | Oxygen-insensitive NADPH-nitroreductase, inhibits anaerobic and protozoal DNA synthesis | 4173 (C_6_H_9_N_3_O_3_) | Hollis et al., 2023; Rasi et al., 2010 |
|  | Miconazole #  (imidazole/ azole derivative) | Antifungal, diaper dermatitis, antibacterial, vaginal yeast infections, etc | Fungal CYP450 14α-lanosterol demethylase and nitric oxide synthase | 4189 (C_18_H_14_Cl_4_N_2_O) | Lodi et al., 2007; Fujita et al., 2023 |
|  | Mycophenolate mofetil ** (mycophenolic acid/ phthalide derivative) | Immunosuppressive, anti-proliferative, prodrug for kidney, cardiac transplants (with corticosteroids), etc. | Inosine monophosphate dehydrogenase 1 and 2, 6-pyruvoyl tetrahydro-biopterin synthase | 5281078 (C_23_H_31_NO_7_) | Dalmau et al., 2007; Samiee et al., 2020; Goel et al., 2021; Wee et al., 2012 |
|  | Nystatin *#  (macrolide class of derivative) | Antifungal (skin, vaginal, mouth, esophageal candida infections, antibacterial, etc. | Ergosterol targeting fungal cell membrane inhibition | 6433272 (C_47_H_75_NO_17_) | Amirchaghmaghi et al., 2015; Rivarola de Gutierrez et al., 2014; Ferri et al., 2018 |
|  | Prednisolone *$  (glucocorticoid/ steroid) | Antiinflammatory, rheumatic arthritis, neoplastic, asthma, allergy, dermatitis, etc. | Glucocorticoid receptor, phospholipase A2 | 5755  (C_21_H_28_O_5_) | Kia et al., 2020; Koszorú et al., 2023; Lu et al., 2019; Kuo et al., 2013 |
|  | Tacrolimus *#  (macrolide lactone) | Immunosuppressive, atopic dermatitis, vitiligo, eczema, psoriasis, used to reduce risk of organ rejection, etc. | Peptidyl-prolyl cis-trans isomerase FKBP1A, transcription factor, IL-3, IL-4, IL-5, and TNF | 445643 (C_44_H_69_NO_12_) | Su et al., 2022; Utz et al., 2022; Shipley and Spivakovsky , 2016; Pinto et al., 2023; Walia et l., 2022; AlMutairi et al., 2023; Kiyani et al., 2021 |
|  | Thalidomide *  (piperidinylisoindole/ phthalimide derivative | Immunomodulatory, skin disorder, multiple myeloma, antiangiogenic, erythema nodosum leprosum, etc. | Protein cereblon, nuclear factor NF-kappa-B, tumor necrosis factor-α, fibroblast growth factor receptor-2 | 5426 (C_13_H_10_N_2_O_4_) | Thongprasom et al., 2013; Yang et al., 2016; Chen et al., 2023; Chen and Wang et al., 2020; Patil et al., 2023 |
|  | Triamcinolone acetonide *** (glucocorticosteroid/ steroid) | Antiinflammatory, allergy, multiple sclerosis, discoid, lichen planus, lupus erythematosus, etc. | Glucocorticoid receptor, phospholipase A2 | 6436 (C_24_H_31_FO_6_) | Zhao et al., 2022; Chaitanya et al., 2022; Walia et al., 2022; Agha-Hosseini et al., 2021; Bajoria et al., 2023; Salinas-Gilabert et al., 2022; Murugan et al., 2023 |

**Note**: *, oral; **, oral with injectable; ***, oral, topical with injectable; #, topical; $, spray/ inhaler. Among all, the serial numbers- 2 (betamethasone), 5 (clobetasol propionate), 8 (dexamethasone), 12 (Fluocinonide), 13 (**fluocinolone acetonide**), 14 (fluticasone), 15 (fluticasone propionate), 25 (prednisolone), 28 (triamcinolone acetonide) are steroid class of drugs and rest of all are non-steroid class of drugs.

**Table S2**. Grid box settings for each target to get a reliable docking score.

| **Target protein (PDB ID)** | **Grid box dimensions (X, Y, Z axis)** | **Grid spacing (Å)** |
| --- | --- | --- |
| **IL1α** (PDB ID: 5UC6) | 108 x 84 x 86 | 0.481 |
| **IL2** (PDB ID: 1M49) | 82 x 84 x 124 | 0.386 |
| **IL4** (PDB ID: 1HIK) | 108 x 76 x 74 | 0.408 |
| **IL6** (PDB ID: 4O9H) | 84 x 84 x 118 | 0.376 |
| **IL10** (PDB ID: 1LQS) | 74 x 122 x 110 | 0.497 |
| **IL17α** (PDB ID: 8CDG) | 98 x 96 x 98 | 0.427 |
| **TNFα1** (PDB ID: 1TNF) | 96 x 96 x 96 | 0.431 |
| **IFNγ3** (PDB ID: 1QWT) | 126 x 114 x 88 | 0.408 |
| **NF_K_B** (PDB ID: 1SVC) | 114 x 88 x 88 | 0.581 |
| **HSP70** (PDB ID: 6FDT) | 116 x 94 x 92 | 0.381 |
| **GR** (PDB ID: 4P6W) | 116 x 94 x 96 | 0.431 |
| **TLR7** (PDB ID: 5GMG) | 126 x 126 x 126 | 0.558 |

**
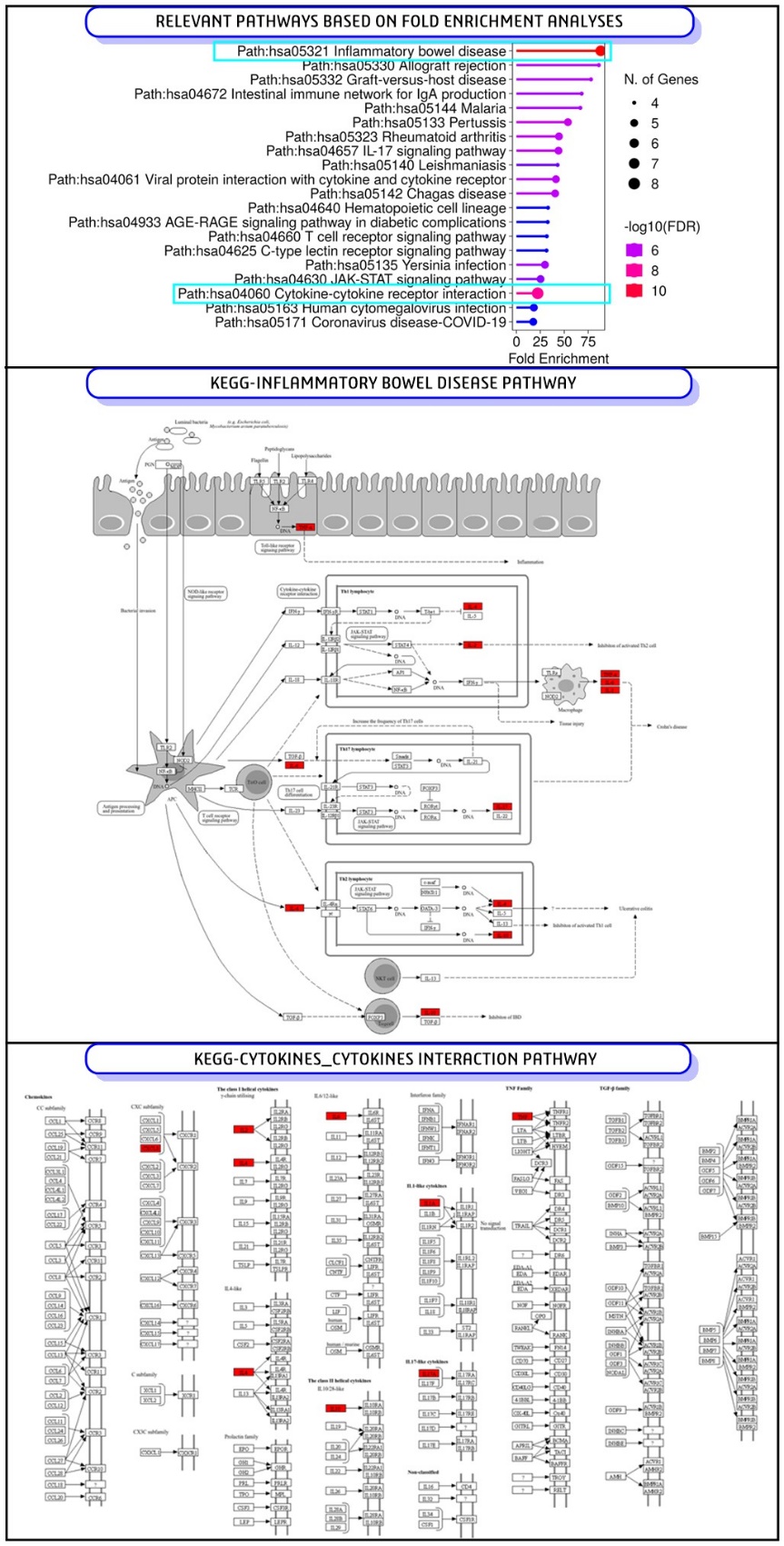
**

**Fig. S1.** Out of twenty predicted pathways, we selected inflammatory bowel diseases and cytokine-cytokine interaction pathways as the two most relevant ones associated with OLP pathophysiology through gene enrichment analyses. Furthermore, the illustrated pathways prominently highlight in red the most probable bioactive target genes.


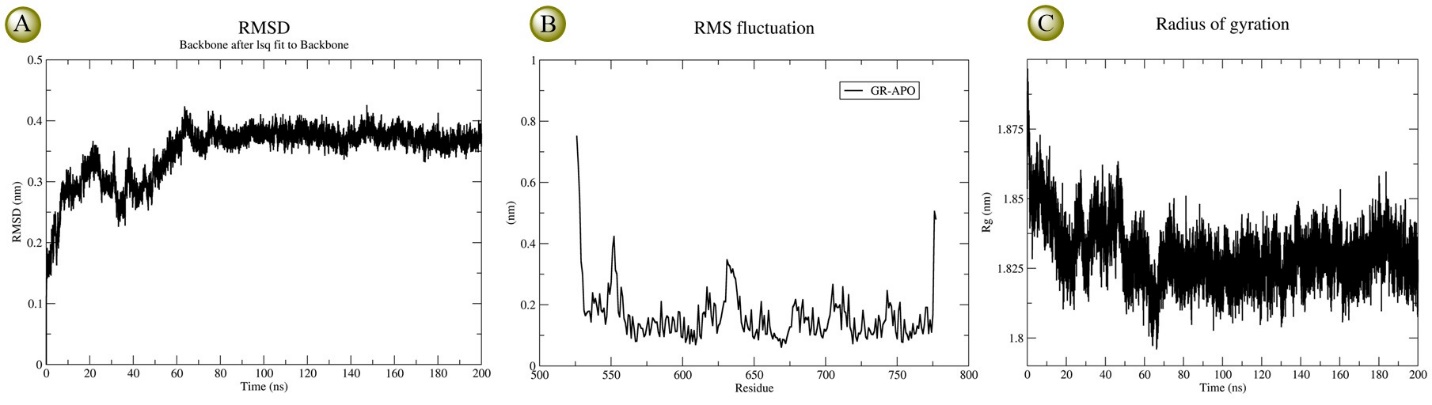


**Fig. S2**. Conformational stability in the form of RMSD, RMSF, and Rg-score plots of apo-protein (GR) at 200 ns; (**A**), RMSD-plots; (**B**), RMSF-plots; and (**C**), Rg-plots, respectively.

**Reference**s (**Table S1**):

1. Didona D, Caposiena Caro RD, Sequeira Santos AM, Solimani F, Hertl M. Therapeutic strategies for oral lichen planus: State of the art and new insights. Front Med (Lausanne). 2022;9:997190. doi: 10.3389/fmed.2022.997190.
2. Verma KK, Mittal R, Manchanda Y. Azathioprine for the treatment of severe erosive oral and generalized lichen planus. Acta Derm Venereol. 2001;81:378-9. doi: 10.1080/000155501317140197.
3. Ezzatt OM, Helmy IM. Topical pimecrolimus versus betamethasone for oral lichen planus: a randomized clinical trial. Clin Oral Investig. 2019;23:947-956. doi: 10.1007/s00784-018-2519-6.
4. Gamal Alsakaan NA, Abd-Elsalam S, Fawzy MM, Elwan NM. Efficacy and safety of oral methotrexate versus oral mini pulse betamethasone therapy in the treatment of lichen planus: a comparative study. J Dermatolog Treat. 2022;33:3039-3046. doi: 10.1080/09546634.2022.2104446.
5. Samimi M, Le Gouge A, Boralevi F, Passeron T, Pascal F, Bernard P, Agbo-Godeau S, Leducq S, Fricain JC, Vaillant L, Francès C. Topical rapamycin versus betamethasone dipropionate ointment for treating oral erosive lichen planus: a randomized, double-blind, controlled study. J Eur Acad Dermatol Venereol. 2020;34(10):2384-2391. doi: 10.1111/jdv.16324.
6. Marable DR, Bowers LM, Stout TL, Stewart CM, Berg KM, Sankar V, DeRossi SS, Thoppay JR, Brennan MT. Oral candidiasis following steroid therapy for oral lichen planus. Oral Dis. 2016;22(2):140-7.
7. Vaishnavi Devi B, Santhosh Kumar MP, Treatment Modalities in the Management of Oral Lichen Planus-An Institutional Experience, J Res Med Dent Sci, 2022, 10 (8): 184-195.
8. Mamadapur R, Naik Z, Kumar SL, Bagewadi A. Comparative efficacy of topical coconut cream and clobetasol propionate ointment for the management of oral lichen planus: A double-blinded randomized control trial. Indian J Pharmacol. 2022;54(2):84-89. doi: 10.4103/ijp.ijp_984_20.
9. Kumar S L, Naik Z, Panwar A, M S, Keluskar V, Kumar RS. Comparative evaluation of the efficacy of Nigella sativa (75% v/v) cream and clobetasol propionate (0.05% w/w) gel in oral lichen planus-a double-blinded randomized control trial. Oral Maxillofac Surg. 2022. doi: 10.1007/s10006-022-01130-6.
10. Arduino PG, Campolongo MG, Sciannameo V, Conrotto D, Gambino A, Cabras M, Ricceri F, Carossa S, Broccoletti R, Carbone M. Randomized, placebo-controlled, double-blind trial of clobetasol propionate 0.05% in the treatment of oral lichen planus. Oral Dis. 2018;24(5):772-777. doi: 10.1111/odi.12821.
11. Brennan MT, Madsen LS, Saunders DP, Napenas JJ, McCreary C, Ni Riordain R, Pedersen AML, Fedele S, Cook RJ, Abdelsayed R, Llopiz MT, Sankar V, Ryan K, Culton DA, Akhlef Y, Castillo F, Fernandez I, Jurge S, Kerr AR, McDuffie C, McGaw T, Mighell A, Sollecito TP, Schlieve T, Carrozzo M, Papas A, Bengtsson T, Al-Hashimi I, Burke L, Burkhart NW, Culshaw S, Desai B, Hansen J, Jensen P, Menné T, Patel PB, Thornhill M, Treister N, Ruzicka T. Efficacy and safety of a novel mucoadhesive clobetasol patch for treatment of erosive oral lichen planus: A phase 2 randomized clinical trial. J Oral Pathol Med. 2022;51(1):86-97. doi: 10.1111/jop.13270.
12. Monshi B, Ellersdorfer C, Edelmayer M, Dvorak G, Ganger C, Ulm C, Rappersberger K, Vujic I. Topical Cyclosporine in Oral Lichen Planus-A Series of 21 Open-Label, Biphasic, Single-Patient Observations. J Clin Med. 2021;10(22):5454. doi: 10.3390/jcm10225454.
13. Georgaki M, Piperi E, Theofilou VI, Pettas E, Stoufi E, Nikitakis NG. A randomized clinical trial of topical dexamethasone vs. cyclosporine treatment for oral lichen planus. Med Oral Patol Oral Cir Bucal. 2022;27(2):e113-e124. doi: 10.4317/medoral.25040.
14. Singh AR, Rai A, Aftab M, Jain S, Singh M. Efficacy of steroidal vs non-steroidal agents in oral lichen planus: a randomised, open-label study. J Laryngol Otol. 2017;131(1):69-76. doi: 10.1017/S0022215116009658.
15. Verma P, Pandhi D. Topical Tacrolimus and Oral Dapsone Combination Regimen in Lichen Planus Pigmentosus. Skinmed. 2015;13(5):351-4.
16. Villa A, Sankar V, Bassani G, Johnson LB, Sroussi H. Dexamethasone solution and dexamethasone in Mucolox for the treatment of oral lichen planus: a preliminary study. Oral Surg Oral Med Oral Pathol Oral Radiol. 2020;129:585-590. doi: 10.1016/j.oooo.2020.02.014.
17. Zhang Z, Jia Y, Tao L, Liu X, Han Y, Wang X. Clinical Evaluation of Dexamethasone Plus Gentamycin Mouthwash Use in Combination with Total Glucosides of Paeony for Treatment of Oral Lichen Planus without Fungal Infection: A Comparative Study with Long-Term Follow-Up. J Clin Med. 2022;11(23):7004. doi: 10.3390/jcm11237004.
18. Hambly JL, Haywood A, Hattingh L, Nair RG. Comparison between self-formulation and compounded-formulation dexamethasone mouth rinse for oral lichen planus: a pilot, randomized, cross-over trial. J Investig Clin Dent. 2017;8(3). doi: 10.1111/jicd.12225.
19. NCT00484250, Study of Metronidazole and Doxycycline to Treat Oral Lichen Planus and to Compare Their Efficacy With Each Other.
20. Arash Mansourian, Majid Sanatkhani and Fatemeh Momen Heravi, 2008. A Randomized Double Blind Controlled Trial to Compare Metronidazole with Doxycycline for the Treatment of Oral Lichen Planus. Journal of Medical Sciences, 8: 201-204.
21. Piacentini M, Borghetti RL, Zancanaro de Figueiredo MA, Cherubini K, Gonçalves Salum F. Doxycycline: An option in the treatment of ulcerated oral lesions? J Clin Pharm Ther. 2019;44(6):838-843. doi: 10.1111/jcpt.13022.
22. Perschy L, Anzengruber F, Rappersberger K, Itzlinger-Monshi B, Aichelburg MC, Graf V, Hafner J, Vujic I. Apremilast in oral lichen planus - a multicentric, retrospective study. J Dtsch Dermatol Ges. 2022;20(3):343-346. doi: 10.1111/ddg.14696.
23. Bettencourt M. Oral Lichen Planus Treated With Apremilast. J Drugs Dermatol. 2016;15(8):1026-8.
24. Kim-Lim P, Thomas C. Crushed apremilast for the treatment of oral lichen planus. JAAD Case Rep. 2023;37:114-115. doi: 10.1016/j.jdcr.2023.05.013.
25. AbuHilal M, Walsh S, Shear N. Treatment of recalcitrant erosive oral lichen planus and desquamative gingivitis with oral apremilast. J Dermatol Case Rep. 2016;10(3):56-57. doi: 10.3315/jdcr.2016.1232.
26. Rotaru D, Chisnoiu R, Picos AM, Picos A, Chisnoiu A. Treatment trends in oral lichen planus and oral lichenoid lesions (Review). Exp Ther Med. 2020;20(6):198.
27. Petruzzi M, Lucchese A, Lajolo C, Campus G, Lauritano D, Serpico R. Topical retinoids in oral lichen planus treatment: an overview. Dermatology. 2013;226(1):61-7. doi: 10.1159/000346750.
28. Davari P, Hsiao HH, Fazel N. Mucosal lichen planus: an evidence-based treatment update. Am J Clin Dermatol. 2014;15(3):181-95.
29. Thongprasom K. A Review of the Effectiveness and Side-Effects of Fluocinolone Acetonide 0.1% in the Treatment of Oral Mucosal Diseases. Acta Stomatol Croat. 2017;51(3):240-247. doi: 10.15644/asc51/3/8.
30. Buajeeb W, Pobrurksa C, Kraivaphan P. Efficacy of fluocinolone acetonide gel in the treatment of oral lichen planus. Oral Surg Oral Med Oral Pathol Oral Radiol Endod. 2000;89(1):42-5. doi: 10.1016/s1079-2104(00)80012-8.
31. Saengprasittichok N, Sucharitakul J, Matangkasombut O, Prapinjumrune C. Effect of fluocinolone acetonide (0.1%) treatment in oral lichen planus patients on salivary lactoferrin levels and Candida colonization: a prospective study. BMC Oral Health. 2022;22(1):58. doi: 10.1186/s12903-022-02096-3.
32. Hegarty AM, Hodgson TA, Lewsey JD, Porter SR. Fluticasone propionate spray and betamethasone sodium phosphate mouthrinse: a randomized crossover study for the treatment of symptomatic oral lichen planus. J Am Acad Dermatol. 2002;47(2):271-9. doi: 10.1067/mjd.2002.120922.
33. Ynson ML, Forouhar F, Vaziri H. Case report and review of esophageal lichen planus treated with fluticasone. World J Gastroenterol. 2013;19(10):1652-6. doi: 10.3748/wjg.v19.i10.1652.
34. F. Donnellan, M. P. Swan, G. R. May, G. Kandel, N. E. Marcon, P. P. Kortan, Fluticasone propionate for treatment of esophageal lichen planus. a case series, Diseases of the Esophagus, 2011;24:211–214, <https://doi.org/10.1111/j.1442-2050.2010.01120.x>
35. Naylor GD. Treating erosive lichen planus with griseofulvin: a report of four cases. Quintessence Int. 1990;21(12):943-7.
36. Matthews RW, Scully C. Griseofulvin in the treatment of oral lichen planus: adverse drug reactions, but little beneficial effect. Ann Dent. 1992;51(2):10-1.
37. Raj SC, Baral D, Garhnayak L, Mahapatra A, Patnaik K, Tabassum S, Dash JK. Hydroxychloroquine- A new treatment option for erosive oral lichen planus. Indian J Dent Res. 2021;32(2):192-198. doi: 10.4103/ijdr.IJDR_943_20.
38. Yeshurun A, Bergman R, Bathish N, Khamaysi Z. Hydroxychloroquine sulphate therapy of erosive oral lichen planus. Australas J Dermatol. 2019;60(2):e109-e112. doi: 10.1111/ajd.12948.
39. Xie Y, Xu H, Li C, Wang Y, Lu R, Hua H, Tang G, Zhou G, Jin X, Shang Q, Dan P, Zhang C, Luo X, Dan H, Zeng X, Zhou Y, Chen Q. Hydroxychloroquine is effective in oral lichen planus: A multicenter, randomized, controlled trial. Oral Dis. 2023. doi: 10.1111/odi.14746.
40. Petruzzi M, Lucchese A, Lajolo C, Campus G, Lauritano D, Serpico R. Topical retinoids in oral lichen planus treatment: an overview. Dermatology. 2013;226(1):61-7. doi: 10.1159/000346750.
41. Won TH, Park SY, Kim BS, Seo PS, Park SD. Levamisole monotherapy for oral lichen planus. Ann Dermatol. 2009;21(3):250-4. doi: 10.5021/ad.2009.21.3.250.
42. Lu SY, Chang TF, Lin CJ. Treatment effectiveness of levamisole plus prednisolone on oral lichen planus patients with emphasis on levamisole-induced agranulocytosis or pancytopenia. J Formos Med Assoc. 2019;118(8):1193-1201. doi: 10.1016/j.jfma.2019.03.007.
43. Chiang CP. Levamisole is an effective immunomodulator for patients with oral lichen planus. J Formos Med Assoc. 2012;111(11):661. doi: 10.1016/j.jfma.2012.09.003.
44. Chauhan P, De D, Handa S, Narang T, Saikia UN. A prospective observational study to compare efficacy of topical triamcinolone acetonide 0.1% oral paste, oral methotrexate, and a combination of topical triamcinolone acetonide 0.1% and oral methotrexate in moderate to severe oral lichen planus. Dermatol Ther. 2018;31(1). doi: 10.1111/dth.12563.
45. Goel S, Khurana N. Effects of Mycophenolate Mofetil, Methotrexate and Pimecrolimus on cdk4 and p16 in Erosive Oral Lichen Planus. Indian J Dermatol. 2021;66:490-495. doi: 10.4103/ijd.IJD_237_17.
46. Lajevardi V, Ghodsi SZ, Hallaji Z, Shafiei Z, Aghazadeh N, Akbari Z. Treatment of erosive oral lichen planus with methotrexate. J Dtsch Dermatol Ges. 2016;14(3):286-93. doi: 10.1111/ddg.12636.
47. Rasi A, Behzadi AH, Davoudi S, Rafizadeh P, Honarbakhsh Y, Mehran M, Piran P, Dehghan N. Efficacy of oral metronidazole in treatment of cutaneous and mucosal lichen planus. J Drugs Dermatol. 2010;9(10):1186-90.
48. Hollis AN, Myers EL, Culton DA. A retrospective cohort study on the efficacy of metronidazole in oral lichen planus. Clin Exp Dermatol. 2023:llad268. doi: 10.1093/ced/llad268.
49. Fujita Y, Sugai T, Maya Y, Inamura E, Hirano Y, Shimizu S. Secukinumab-induced oral lichen planus in a psoriatic arthritis patient ameliorated after a switch to risankizumab. J Dermatol. 2023;50(6):824-827. doi: 10.1111/1346-8138.16719.
50. Lodi G, Tarozzi M, Sardella A, Demarosi F, Canegallo L, Di Benedetto D, Carrassi A. Miconazole as adjuvant therapy for oral lichen planus: a double-blind randomized controlled trial. Br J Dermatol. 2007;156(6):1336-41. doi: 10.1111/j.1365-2133.2007.07883.x.
51. Wee JS, Shirlaw PJ, Challacombe SJ, Setterfield JF. Efficacy of mycophenolate mofetil in severe mucocutaneous lichen planus: a retrospective review of 10 patients. Br J Dermatol. 2012;167(1):36-43. doi: 10.1111/j.1365-2133.2012.10882.x.
52. Samiee N, Taghavi Zenuz A, Mehdipour M, Shokri J. Treatment of oral lichen planus with mucoadhesive mycophenolate mofetil patch: A randomized clinical trial. Clin Exp Dent Res. 2020;6(5):506-511. doi: 10.1002/cre2.302.
53. Goel S, Khurana N. Effects of Mycophenolate Mofetil, Methotrexate and Pimecrolimus on cdk4 and p16 in Erosive Oral Lichen Planus. Indian J Dermatol. 2021;66(5):490-495. doi: 10.4103/ijd.IJD_237_17.
54. Dalmau J, Puig L, Roé E, Peramiquel L, Campos M, Alomar A. Successful treatment of oral erosive lichen planus with mycophenolate mofetil. J Eur Acad Dermatol Venereol. 2007;21(2):259-60. doi: 10.1111/j.1468-3083.2006.01832.x.
55. Ferri EP, Gallo CB, Abboud CS, Yanaguizawa WH, Horliana ACRT, Silva DFTD, Pavani C, Bussadori SK, Nunes FD, Mesquita-Ferrari RA, Fernandes KPS, Rodrigues MFSD. Efficacy of photobiomodulation on oral lichen planus: a protocol study for a double-blind, randomised controlled clinical trial. BMJ Open. 2018;8(10):e024083. doi: 10.1136/bmjopen-2018-024083.
56. Rivarola de Gutierrez E, Di Fabio A, Salomón S, Lanfranchi H. Topical treatment of oral lichen planus with anthocyanins. Med Oral Patol Oral Cir Bucal. 2014;19(5):e459-66. doi: 10.4317/medoral.19472.
57. Amirchaghmaghi M, Delavarian Z, Iranshahi M, Shakeri MT, Mosannen Mozafari P, Mohammadpour AH, Farazi F, Iranshahy M. A Randomized Placebo-controlled Double Blind Clinical Trial of Quercetin for Treatment of Oral Lichen Planus. J Dent Res Dent Clin Dent Prospects. 2015;9(1):23-8. doi: 10.15171/joddd.2015.005.
58. Kuo RC, Lin HP, Sun A, Wang YP. Prompt healing of erosive oral lichen planus lesion after combined corticosteroid treatment with locally injected triamcinolone acetonide plus oral prednisolone. J Formos Med Assoc. 2013;112(4):216-20. doi: 10.1016/j.jfma.2012.01.014.
59. Lu SY, Chang TF, Lin CJ. Treatment effectiveness of levamisole plus prednisolone on oral lichen planus patients with emphasis on levamisole-induced agranulocytosis or pancytopenia. J Formos Med Assoc. 2019;118(8):1193-1201. doi: 10.1016/j.jfma.2019.03.007.
60. Koszorú K, Kovács A, Lőrincz K, Medvecz M, Sárdy M. Low dose oral glucocorticoid therapy in lichen planus: A retrospective cohort study. Indian J Dermatol Venereol Leprol. 2023;89(4):568-571. doi: 10.25259/IJDVL_1111_2021.
61. Kia SJ, Basirat M, Mortezaie T, Moosavi MS. Comparison of oral Nano-Curcumin with oral prednisolone on oral lichen planus: a randomized double-blinded clinical trial. BMC Complement Med Ther. 2020;20(1):328. doi: 10.1186/s12906-020-03128-7.
62. Kiyani A, Sohail K, Saeed MHB. Efficacy of 0.1% tacrolimus in long-term management of erosive lichen planus. J Dermatolog Treat. 2021;32(3):367-371. doi: 10.1080/09546634.2019.1654072.
63. AlMutairi M, Riyaz SMA, Awinashe M, Almutairi FJ. Assessment of 5% Amlexanox, 0.1% Triamcinolone Acetonide and 0.03% Tacrolimus in the Management of Oral Lichen Planus. J Pharm Bioallied Sci. 2023;15(Suppl 2):S1298-S1300. doi: 10.4103/jpbs.jpbs_112_23.
64. Walia C, Rallan NS, Premkumar A, Roy S. Clinical Evaluation of Efficacy of Triamcinolone Acetonide with Tacrolimus in the Management of Oral Lichen Planus: A Pilot Prospective Observational Study. Contemp Clin Dent. 2022;13(3):236-241. doi: 10.4103/ccd.ccd_899_20.
65. Pinto J, Waghmare M, Bhor K, Santosh V, Manoj R, Samson S. Efficacy and Safety of Topical Tacrolimus in Comparison with Topical Corticosteroids, Calcineurin Inhibitors, Retinoids and Placebo in Oral Lichen Planus: An Updated Systematic Review and Meta-Analysis. Asian Pac J Cancer Prev. 2023;24(2):389-400. doi: 10.31557/APJCP.2023.24.2.389.
66. Utz S, Suter VGA, Cazzaniga S, Borradori L, Feldmeyer L. Outcome and long-term treatment protocol for topical tacrolimus in oral lichen planus. J Eur Acad Dermatol Venereol. 2022;36(12):2459-2465. doi: 10.1111/jdv.18457.
67. Su Z, Hu J, Cheng B, Tao X. Efficacy and safety of topical administration of tacrolimus in oral lichen planus: An updated systematic review and meta-analysis of randomized controlled trials. J Oral Pathol Med. 2022;51(1):63-73. doi: 10.1111/jop.13217.
68. Patil S, Mustaq S, Hosmani J, Khan ZA, Yadalam PK, Ahmed ZH, Bhandi S, Awan KH. Advancement in therapeutic strategies for immune-mediated oral diseases. Dis Mon. 2023;69(1):101352. doi: 10.1016/j.disamonth.2022.101352.
69. Chen X, Wang S. Case of erythrodermic lichen planus successful treated with thalidomide monotherapy. J Dermatol. 2020;47(6):e224-e225. doi: 10.1111/1346-8138.15332.
70. Chen N, Tian Y, Wu L, Wang H, Liang X, Zhou H. Successful treatment of refractory oral lichen planus with microwave therapy. Australas J Dermatol. 2023;64(1):150-152. doi: 10.1111/ajd.13973.
71. Yang H, Wu Y, Ma H, Jiang L, Zeng X, Dan H, Zhou Y, Chen Q. Possible alternative therapies for oral lichen planus cases refractory to steroid therapies. Oral Surg Oral Med Oral Pathol Oral Radiol. 2016;121(5):496-509. doi: 10.1016/j.oooo.2016.02.002.
72. Thongprasom K, Prapinjumrune C, Carrozzo M. Novel therapies for oral lichen planus. J Oral Pathol Med. 2013;42(10):721-7. doi: 10.1111/jop.12083.
73. Zhao W, Lin D, Deng S, Wang S, Guo Y, Yang J, Shi X, Zhou H. Synergistic Efficacy of Plaque Control with Intralesional Triamcinolone Acetonide Injection on Erosive Non-Gingival Oral Lichen Planus: A Randomized Controlled Clinical Trial. Int J Environ Res Public Health. 2022;19(21):13787. doi: 10.3390/ijerph192113787.
74. Chaitanya NC, Chikte D, Kumar YP, Komali G, Yellarthi SP, Reddy CS, Harika DP, Haritha S, Taie WAA, Hatab NA, Patil S, Panta P. Efficacy of Spirulina 500 mg vs Triamcinolone Acetonide 0.1% for the Treatment of Oral Lichen Planus: A Randomized Clinical Trial. J Contemp Dent Pract. 2022;23(5):552-557.
75. Walia C, Rallan NS, Premkumar A, Roy S. Clinical Evaluation of Efficacy of Triamcinolone Acetonide with Tacrolimus in the Management of Oral Lichen Planus: A Pilot Prospective Observational Study. Contemp Clin Dent. 2022;13(3):236-241. doi: 10.4103/ccd.ccd_899_20.
76. Agha-Hosseini F, Atyabi F, Akbari K, Moosavi MS. Decreased recurrence of symptoms in oral lichen planus with intralesional injection of hyaluronic acid and triamcinolone. Int J Oral Maxillofac Surg. 2021;50(12):1643-1648. doi: 10.1016/j.ijom.2021.02.028.
77. Bajoria AA, Chinnannavar SN, Mishra S, Singh DK, Pathi J, Jha VK. Comparative Evaluation of Pimecrolimus Cream 1% and Triamcinolone Aceonide Paste in Treatment of Atrophic-Erosive Oral Lichen Planus. J Pharm Bioallied Sci. 2023;15:S1274-S1276. doi: 10.4103/jpbs.jpbs_127_23.
78. Salinas-Gilabert C, Gómez García F, Galera Molero F, Pons-Fuster E, Vander Beken S, Lopez Jornet P. Photodynamic Therapy, Photobiomodulation and Acetonide Triamcinolone 0.1% in the Treatment of Oral Lichen Planus: A Randomized Clinical Trial. Pharmaceutics. 2022;15(1):30. doi: 10.3390/pharmaceutics15010030.
79. Murugan AJ, Ganesan A, Aniyan YK, Lakshmi KC, Asokan K. Comparison of topical purslane & topical 0.1% triamcinolone acetonide in the management of oral lichen planus - a double blinded clinical trial. BMC Oral Health. 2023;23(1):678. doi: 10.1186/s12903-023-03385-1.
